# A Structure Shaped by Fire, but Also Water: Ecological Consequences of the Variability in Bark Properties Across 31 Species From the Brazilian Cerrado

**DOI:** 10.3389/fpls.2019.01718

**Published:** 2020-01-22

**Authors:** Lucas Loram-Lourenço, Fernanda dos Santos Farnese, Letícia Ferreira de Sousa, Rauander Douglas Ferreira Barros Alves, Maria Clara Pereira de Andrade, Sabrina Emanuella da Silva Almeida, Luciana Minervina de Freitas Moura, Alan Carlos Costa, Fabiano Guimarães Silva, Jeroni Galmés, Hervé Cochard, Augusto Cesar Franco, Paulo Eduardo Menezes-Silva

**Affiliations:** ^1^ Departamento de Biologia, Instituto Federal de Educação, Ciência e Tecnologia Goiano, Campus Rio Verde, Rio Verde, Brazil; ^2^ Research Group on Plant Biology Under Mediterranean Conditions, Department of Biology, Universitat de les Illes Balears, Palma, Spain; ^3^ Université Clermont-Auvergne, INRA, PIAF, Clermont-Ferrand, France; ^4^ Departamento de Botânica, Instituto de Ciências Biológicas, Universidade de Brasília, Brasília, Brazil

**Keywords:** bark ecophysiology, cerrado, fire resistance, water storage and transport, plant functional traits

## Abstract

Bark is a structure involved in multiple physiological functions, but which has been traditionally associated with protection against fire. Thus, little is known about how the morpho-anatomical variations of this structure are related to different ecological pressures, especially in tropical savanna species, which are commonly subjected to frequent fire and drought events. Here we evaluated how the structural and functional variations of bark are related to the processes of resilience and resistance to fire, as well as transport and storage of water in 31 native species from the Brazilian Cerrado. Because of their thick bark, none of the trees analyzed were top-killed after a severe fire event. The structural and functional variations of the bark were also associated with water storage and transport, functions related to properties of the inner bark. In fact, species with a thicker and less dense inner bark were the ones that had the highest water contents in the wood, bark, and leaves. Lower bark density was also related to higher stem hydraulic conductivity, carbon assimilation, and growth. Overall, we provide strong evidence that in addition to protection from fire, the relative investment in bark also reflects different strategies of water use and conservation among many Cerrado tree species.

## Introduction

Under natural conditions, plants are exposed to a multitude of potentially stressful biotic and abiotic factors that act as important selective pressures with the potential to shape different aspects of their morphology. In fact, some studies have already shown that structural variation in plant organs may reflect different ecological strategies among species of the same community or of different biomes (Chave et al., 2009; Osnas et al., 2013; Sakschewski et al., 2015; Kunstler et al., 2016). Among these structures, one of paramount importance is the bark, a stem component that assumes primordial mechanical and physiological functions, and which has been suggested to be highly plastic in relation to different environmental factors (Rosell et al., 2014; Pausas, 2015a; Rosell, 2016; Gauthier and Guillemette, 2018). However, despite the significant structural variability found among different species, the ecological consequences of the variation in bark components remain unclear (Poorter et al., 2014; Rosell et al., 2017).

Bark is defined as the set of tissues external to the vascular cambium and is structurally divided into two distinct parts: the outer bark (OB) and the inner bark (IB) (Lawes et al., 2013; Rosell et al., 2017). The OB is composed of dead cells and provides structural support and protection against mechanical damage and pathogen attack. The IB, in turn, is formed by living tissues, including the phloem, which is responsible for the storage and transport of water and solutes throughout the plant (Rosell et al., 2017). Among the various ecological functions assumed by bark, perhaps the most studied is the protection against fire. Several studies have demonstrated that some structural properties of the bark are determinants in the survival of species inhabiting regions exposed to frequent fires such as savannas (Hoffmann et al., 2009; Lawes et al., 2013; Pausas, 2015a; Rosell, 2016). The protective capacity of bark against fire is due to its excellent insulating properties, which prevent irreversible damage to the vascular cambium (Graves et al., 2014). This insulating effect, in turn, increases significantly with the thickness of the bark, such that this trait has been widely suggested as one of the best predictors of the survival rates of plant species after fire events (Hoffmann et al., 2009; Lawes et al., 2013; Pellissier et al., 2013; Pellegrini et al., 2017). Besides protecting the stem meristematic cells, the bark structural properties also seem to be important in protecting the hydraulic system from damages induced by fire. In fact, it was already showed that a combination of bark (e.g., high thickness) and wood traits (hydraulic segmentation) can avoid xylem deformation during fire episodes, which may increase recovery, and thus avoid drought-induced tree mortality after fire events (West et al., 2016).

In addition to protection against fire, which is a function primarily exerted by the OB, some evidence suggests that the IB can also assume ecological functions of paramount importance (Rosell et al., 2014). One of these functions involves water storage and the maintenance of water transport, both daily and seasonally. It has been shown that water stored in the bark is fundamental for the regulation of water status throughout the day, and also for the supply of water to emerging leaves, particularly in tropical regions where regrowth occurs after periods of drought (Scholz et al., 2007; Poorter et al., 2014). In this sense, the bark has been described as a large capacitor (Pfautsch et al., 2015), which would be related to the maintenance and regulation of water transport throughout the plant and, therefore, plays a central role in water relations (Poorter et al., 2014; Rosell et al., 2014; Mason Earles et al., 2016). Because of the presence of the phloem, the IB is also involved in the transport of photosynthates and other substances, which reinforces the importance of this structure in the maintenance of central physiological processes (Rosell et al., 2014; Rosell et al., 2017). Thus, in order to sustain their water and carbohydrates demands, it is expected that species with different growth strategies (e.g., contrasting growth rates) may differ significantly in their relative investment in IB.

The role of bark in the regulation of several aspects of water relations and carbohydrate transport suggests that in addition to the selective pressure exerted by fire, variation in this structure among species can also be the result of different strategies of water use and conservation, besides reflecting contrasting metabolic demands (Poorter et al., 2014; Rosell et al., 2014). Consequently, species that inhabit regions subject to these two selective pressures (fire and drought) are expected to exhibit significant structural and functional variation in bark components. This may be particularly true for plants that inhabit the Cerrado, the second-largest Brazilian biome and one of the world's largest biodiversity hotspots (Lapola, 2007; Strassburg et al., 2017). The climate of the Cerrado is characterized by a long period of water restriction, approximately 5 months, during which the occurrence of fires is frequent (Hoffmann et al., 2009). This combination of fire and drought was possibly a determining selective factor that shaped several tree lineages that successfully invaded savanna environments from adjacent forests in the Brazilian Cerrado, which differ significantly in their relative investments in wood and bark from their forest counterparts (Scholz et al., 2007; Hoffmann et al., 2009). However, it is important to highlight that, to date, the great majority of studies related to bark in Cerrado plants had as main focus only the adjustments induced by fire, more specifically at the outer portion of this structure (Hoffmann et al., 2009; Pausas, 2015a; Schafer et al., 2015). Thus, little is known about what impacts the structural variation in the IB can exert on other central physiological aspects, such as water transport and carbon assimilation, particularly among the species with different growth strategies present in this biome.

The main objective of the present study was to evaluate how variations in the structural and functional properties of bark components, especially the inner portion of this structure, are distributed along axis of variation in a multivariate trait space defining plant functional typologies for 31 tree species of the Brazilian savanna (Cerrado). As a reflection of their distinct ontogenetic origin and composition, we expected that the inner (IB) and outer (OB) bark would fulfill contrasting ecological functions: due to the presence of cells related to water storage, variations in the relative investment in IB would be associated with water storage capacity, whereas variations in OB would be more strongly related to mechanical support and/or fire resistance. In this regard, the potential of the IB to store water would scale positively with the amount of the invested tissue (relative thickness) and negatively with the density of that tissue. We also expected a covariation between phloem (IB) and xylem densities, which would reflect the variability in water transport capacity (e.g., stem hydraulic conductivity) across Cerrado tree species. As a result of its multiple physiological functions, variations in the IB structure integrate contrasting strategies of water use and conservation and thus have a direct impact on the process of carbon assimilation and growth among Cerrado tree species. In this regard, in order to sustain their higher water and carbohydrates demands, fast-growing species would have a thicker and less dense IB than slow-growing species.

## Materials and Methods

### Study Site, Species Selection, and Sample Collection

To understand the ecological consequences of the structural variation in the bark components of Cerrado plants, 31 representative species of the tree layer of this biome were sampled. All samplings and analyses were performed on a natural population of a Cerrado fragment belonging to the ecological reserve of the University of Rio Verde, Rio Verde, Goiás (GO), Brazil (17°47'09.2” S 50°57'50.63” W). The mean annual precipitation in the reserve is 1,700 mm, with the dry season extending from May to September, and the mean annual temperature is 23 °C. The species selected were the most abundant in the region, and five adult plants of each species were analyzed. The selected species cover a great diversity of families (**Table 1**) and encompass substantial structural and functional variability (**Tables 2** and **3**), which ensured a broad representation of the Cerrado tree flora.

**Table 1:** List of species studied.

Species	Family
*Alibertia edulis*	Rubiaceae
*Anacardium occidentale*	Anacardiaceae
*Annona coriacea*	Annonaceae
*Brosimum gaudichaudii*	Moraceae
*Byrsonima basiloba*	Malpighiaceae
*Byrsonima intermedia*	Malpighiaceae
*Caryocar brasiliensis*	Caryocaraceae
*Casearia sylvestris*	Salicaceae
*Ceiba speciosa*	Malvaceae
*Chrysophyllum marginatum*	Sapotaceae
*Curatella americana*	Dilleniaceae
*Diospyros hispida*	Ebenaceae
*Dipteryx alata*	Fabaceae
*Eugenia dysenterica*	Myrtaceae
*Handroanthus albus*	Bignoniaceae
*Handroanthus impetiginosus*	Bignoniaceae
*Handroanthus ochraceae*	Bignoniaceae
*Handroanthus roseoalbus*	Bignoniaceae
*Hymenaea courbaril*	Fabaceae
*Kielmeyera speciosa*	Calophyllaceae
*Machaerium opacum*	Fabaceae
*Myracrodruon urundeuva*	Anacardiaceae
*Pouteria gardneriana*	Sapotaceae
*Qualea parviflora*	Vochysiaceae
*Roupala montana*	Proteaceae
*Spondias mombin*	Anacardiaceae
*Sterculia striata*	Malvaceae
*Terminalia argentea*	Combretaceae
*Tocoyena formosa*	Rubiaceae
*Xylopia aromatica*	Annonaceae
*Zanthoxylum rigidum*	Rutaceae

**Table 2:** Branch-level structural properties of bark and wood from 31 tree species from Brazilian Cerrado.

Species	RBT_total_	RBT_outer_	RBT_inner_	SD	*D*_bark_	*D*_wood_
	%	%	%	cm	g cm^-3^	g cm^-3^
*Annona coriacea*	51.7 (2.28)	2.56 (0.18)	49.1 (2.39)	1.19 (0.04)	0.27 (0.00)	0.30 (0.00)
*Alibertia edulis*	19.6 (0.82)	5.20 (0.80)	14.3 (1.01)	0.95 (0.05)	0.45 (0.01)	0.67 (0.02)
*Anacardium occidentale*	28.8 (1.45)	3.68 (0.68)	25.1 (1.47)	1.09 (0.04)	0.36 (0.00)	0.41 (0.03)
*Brosimum gaudichaudii*	31.2 (2.06)	4.81 (1.39)	26.4 (2.29)	1.13 (0.04)	0.31 (0.02)	0.42 (0.03)
*Byrsonima basiloba*	39.9 (3.67)	11.4 (2.58)	28.4 (1.46)	1.25 (0.12)	0.28 (0.03)	0.42 (0.01)
*Byrsonima intermedia*	38.5 (1.79)	5.49 (0.70)	33.0 (2.06)	1.23 (0.06)	0.37 (0.01)	0.47 (0.01)
*Caryocar brasiliensis*	25.1 (1.26)	2.67 (0.24)	13.0 (0.95)	1.33 (0.06)	0.28 (0.01)	0.35 (0.04)
*Caseria sylvestris*	34.2 (1.69)	10.9 (1.41)	23.1 (0.29)	1.06 (0.11)	0.25 (0.00)	0.46 (0.03)
*Ceiba speciosa*	34.7 (1.42)	3.06 (0.23)	24.1 (4.94)	1.02 (0.02)	0.28 (0.01)	0.42 (0.02)
*Chrysophyllum marginatum*	17.2 (0.20)	4.35 (0.46)	12.8 (0.52)	1.03 (0.07)	0.27 (0.02)	0.42 (0.04)
*Curatella americana*	19.8 (0.61)	4.60 (0.53)	20.6 (2.07)	0.86 (0.04)	0.41 (0.03)	0.37 (0.02)
*Diospyros hispida*	40.2 (0.98)	18.8 (1.09)	21.4 (2.07)	1.52 (0.08)	0.25 (0.01)	0.37 (0.01)
*Dipteryx alata*	21.7 (0.63)	2.87 (0.22)	18.8 (0.87)	0.98 (0.04)	0.46 (0.02)	0.67 (0.04)
*Handroanthus albus*	27.8 (1.44)	7.36 (0.73)	20.4 (0.78)	0.76 (0.03)	0.50 (0.01)	0.64 (0.01)
*Handroanthus impetiginosus*	21.3 (1.55)	3.30 (0.20)	18.0 (1.58)	0.98 (0.04)	0.42 (0.01)	0.56 (0.02)
*Handroanthus ochraceae*	40.4 (1.58)	14.2 (3.01)	26.1 (1.24)	1.41 (0.06)	0.35 (0.03)	0.62 (0.02)
*Handroanthus rosealbus*	24.2 (2.29)	5.17 (0.47)	19.0 (1.97)	0.85 (0.04)	0.45 (0.01)	0.52 (0.01)
*Hymenaeae courbaril*	21.4 (1.55)	5.82 (0.31)	15.5 (1.27)	0.86 (0.05)	0.53 (0.02)	0.56 (0.00)
*Kielmeyera speciosa*	48.3 (2.89)	6.45 (0.87)	41.8 (2.41)	1.32 (0.04)	0.19 (0.00)	0.37 (0.01)
*Machaerium opacum*	41.6 (2.35)	16.0 (3.86)	25.6 (1.51)	1.41 (0.06)	0.38 (0.01)	0.53 (0.01)
*Myracrodruon urundeuva*	19.1 (0.68)	3.17 (0.19)	15.9 (0.61)	0.88 (0.02)	0.42 (0.03)	0.50 (0.03)
*Pouteria gardneriana*	23.6(1.56)	5.58 (0.34)	18.0 (1.43)	0.95 (0.04)	0.35 (0.01)	0.59 (0.03)
*Qualea parviflora*	18.4 (1.35)	6.39 (0.81)	11.9 (1.00)	0.74 (0.04)	0.39 (0.06)	0.51 (0.03)
*Roupala montana*	27.2 (2.06)	5.54 (0.34)	21.7 (1.95)	0.85 (0.02)	0.51 (0.02)	0.51 (0.01)
*Spondias mombin*	20.9 (1.24)	2.36 (0.20)	18.5 (1.05)	1.04 (0.03)	0.31 (0.01)	0.36 (0.02)
*Stenocalyx dysenterica*	17.0 (1.83)	2.32 (0.20)	14.6 (1.64)	0.78 (0.03)	0.56 (0.04)	0.71 (0.02)
*Sterculia striata*	42.1 (1.25)	4.04 (0.39)	24.1 (3.72)	1.27 (0.04)	0.31 (0.02)	0.48 (0.03)
*Terminalia argentea*	29.5 (1.61)	6.36 (0.59)	23.1 (1.80)	0.99 (0.04)	0.40 (0.02)	0.68 (0.02)
*Tocoyena formosa*	24.8 (3.02)	3.60 (0.27)	21.3 (2.75)	1.08 (0.09)	0.36 (0.04)	0.56 (0.03)
*Xylopia aromatica*	27.4 (1.16)	2.69 (0.14)	24.7 (1.02)	0.80 (0.02)	0.36 (0.01)	0.49 (0.03)
*Zanthoxylum rigidum*	19.6 (1.63)	4.24 (0.76)	15.3 (0.87)	1.11 (0.03)	0.38 (0.04)	0.43 (0.04)

Mean ± SE (n = 5) of the relative total bark thickness (RBT_total_), relative outer bark thickness (RBT_outer_), relative inner bark thickness (RBT_inner_), stem diameter (SD), bark density (D_bark_), and wood density (D_wood_).

**Table 3:** Branch-level water relations and growth traits for 31 tree species from Brazilian Cerrado.

Species	WinB	BWC	WWC	LWC	Ψ_stem-md_	*K_stem_*	*A*	BGR
	%	%	%	%	MPa	kg m^-1^ s^-1^ MPa^-1^	µmol g^-1^ s^-1^	mm day^-1^
*Annona coriacea*	51.9 (0.34)	71.3 (0.17)	66.0 (0.92)	62.5 (0.78)	-0.25 (0.01)	4.22 (0.17)	149.3 (1.8)	0.07 (0.01)
*Alibertia edulis*	54.2 (1.10)	51.0 (0.57)	43.2 (1.50)	53.7 (1.35)	-0.88 (0.02)	0.56 (0.11)	77.3 (6.27)	0.13 (0.02)
*Anacardium occidentale*	48.2 (1.54)	67.4 (0.41)	58.2 (2.53)	56.4 (0.40)	-0.20 (0.01)	3.41 (0.31)	158.2 (17.1)	0.20 (0.01)
*Brosimum gaudichaudii*	55.8 (1.09)	69.8 (1.83)	55.6 (2.89)	57.2 (0.52)	-0.14 (0.00)	5.27 (0.09)	222.4 (20.1)	0.09 (0.01)
*Byrsonima basiloba*	51.7 (0.17)	65.9 (1.09)	61.4 (0.88)	56.2 (0.97)	-0.25 (0.01)	5.22 (0.31)	283.7 (28.3)	0.19 (0.03)
*Byrsonima intermedia*	52.1 (0.58)	63.4 (0.97)	58.2 (0.94)	54.4 (0.82)	-0.36 (0.01)	1.6 (0.21)	106.5 (6.83)	0.24 (0.02)
*Caryocar brasiliensis*	42.7 (1.22)	72.5 (0.68)	65.1 (2.24)	57.9 (1.34)	-0.25 (0.00)	5.6 (1.11)	211.1 (8.43)	0.29 (0.11)
*Caseria sylvestris*	55.3 (0.92)	71.1 (0.69)	57.6 (2.46)	59.3 (3.43)	-0.56 (0.01)	3.05 (0.15)	142 (20.0)	0.21 (0.01)
*Ceiba speciosa*	61.3 (1.24)	73.1 (1.08)	53.7 (1.06)	65.4 (0.90)	-1.08 (0.08)	3.88 (0.13)	73.5 (6.42)	0.14 (0.02)
*Chrysophyllum marginatum*	30.3 (1.16)	73.9 (2.12)	57.5 (3.64)	65.6 (2.04)	-0.44 (0.01)	5.22 (0.19)	99.1 (8.20)	0.16 (0.01)
*Curatella americana*	25.6 (1.38)	58.3 (2.99)	66.0 (1.78)	61.1 (0.83)	-0.49 (0.01)	2.11 (0.19)	162.5 (11.4)	0.18 (0.01)
*Diospyros hispida*	49.7 (0.55)	62.1 (1.67)	62.7 (1.01)	59.8 (1.17)	-0.27 (0.02)	2.70 (0.38)	163.6 (2.75)	0.15 (0.01)
*Dipteryx alata*	43.9 (1.24)	58.5 (1.90)	44.0 (2.42)	55.2 (4.25)	-1.18 (0.03)	0.64 (0.08)	53.8 (6.35)	0.07 (0.01)
*Handroanthus albus*	47.7 (1.29)	51.0 (0.65)	43.2 (1.23)	50.7 (0.96)	-0.54 (0.01)	1.38 (0.16)	79.1 (11.7)	0.06 (0.01)
*Handroanthus impetiginosus*	39.9 (2.42)	61.5 (0.9)	49.7 (1.56)	56.4 (0.94)	-0.86 (0.01)	2.10 (0.35)	123.8 (23.5)	0.09 (0.01)
*Handroanthus ochraceae*	54.2 (1.97)	53.5 (4.06)	44.1 (0.58)	53.8 (1.85)	-0.57 (0.01)	1.27 (0.21)	188.0 (28.9)	0.11 (0.03)
*Handroanthus rose-albus*	51.6 (1.03)	60.4 (0.38)	52.3 (1.26)	55.4 (1.60)	-0.80 (0.06)	1.57 (0.10)	111.7 (10.4)	0.05 (0.01)
*Hymenaeae courbaril*	40.3 (1.97)	53.5 (1.25)	43.7 (1.17)	50.7 (0.95)	-1.10 (0.08)	1.74 (0.24)	100.9 (6.92)	0.05 (0.01)
*Kielmeyera speciosa*	55.0 (0.74)	77.3 (0.49)	63.1 (1.48)	65.7 (0.63)	-0.17 (0.00)	3.49 (0.10)	157.4 (14.5)	0.07 (0.01)
*Machaerium opacum*	56.4 (1.41)	60.7 (2.58)	46.6 (0.75)	53.5 (0.45)	-0.30 (0.01)	0.48 (0.13)	72.6 (13.9)	0.29 (0.02)
*Myracrodruon urundeuva*	30.0 (0.79)	63.6 (1.89)	50.3 (2.29)	60.9 (1.84)	-0.16 (0.01)	2.20 (0.16)	72.2 (7.16)	0.06 (0.01)
*Pouteria gardneriana*	39.8 (3.46)	66.4 (1.99)	49.0 (1.93)	56.9 (1.17)	-0.33 (0.01)	2.85 (0.21)	119.9 (10.0)	0.15 (0.01)
*Qualea parviflora*	27.3 (2.12)	67.5 (0.93)	52.3 (1.35)	67.3 (0.28)	-0.86 (0.01)	1.89 (0.11)	77.0 (12.7)	0.08 (0.01)
*Roupala montana*	38.3 (0.81)	53.5 (0.75)	54.2 (1.00)	57.3 (1.19)	-0.43 (0.01)	4.58 (0.42)	120.5 (4.82)	0.04 (0.00)
*Spondias mombin*	44.2 (0.67)	71.1 (0.81)	61.4 (1.18)	65.1 (0.83)	-0.22 (0.01)	6.36 (0.42)	124.5 (11.4)	0.10 (0.01)
*Stenocalyx dysenterica*	29.0 (2.17)	58.7 (1.81)	40.3 (1.52)	45.9 (1.01)	-1.20 (0.03)	2.11 (0.27)	127.2 (9.94)	0.06 (0.01)
*Sterculia striata*	65.8 (1.41)	70.4 (1.61)	54.9 (2.19)	57.0 (0.78)	-0.25 (0.00)	4.00 (0.24)	137.9 (11.4)	0.11 (0.01)
*Terminalia argentea*	58.8 (0.19)	58.8 (1.18)	41.2 (0.81)	54.1 (0.41)	-0.54 (0.03)	0.97 (0.08)	116.4 (18.0)	0.11 (0.01)
*Tocoyena formosa*	58.4 (0.57)	69.4 (1.25)	49.5 (1.91)	56.1 (0.75)	-0.30 (0.01)	1.56 (0.08)	78.7 (11.5)	0.28 (0.04)
*Xylopia aromatica*	53.5 (0.97)	68.0 (1.08)	47.8 (2.16)	57.0 (0.42)	-0.36 (0.01)	2.91 (0.11)	80.7 (18.4)	0.34 (0.01)
*Zanthoxylum rigidum*	40.4 (1.15)	64.8 (2.88)	52.6 (1.96)	60.2 (1.19)	-1.07 (0.06)	3.26 (0.37)	140.2 (9.79)	0.20 (0.03)

Mean ± SE (n = 5) of water in bark (%WinB), bark water content (BWC), wood water content (WWC), leaf water content (LWC), midday leaf water potential (Ψ_leaf-md_), midday stem water potential (Ψ_stem-md_), stem hydraulic conductivity (K_stem_), maximum CO_2_ assimilation on a leaf mass basis (A_mass_), branch growth rate (BGR).

To investigate the relationship between the structural variation in bark components with traits associated with the transport and storage of water, a sun-exposed terminal branch approximately 1 m in length was collected from five individuals per species and used for all morpho-physiological analyses. The bark of the selected branches presented similar morphology to the bark of main trunks (i.e., with OB already developed). This approach provides a clearer picture of the allocation of resources between bark and wood and allows a more in-depth analysis of the interrelationship between structural and functional variation among different tissues (Rosell et al., 2014). All the morphological and physiological analyses were conducted during the rainy season between the months of February and April.

### Fire Survival

**Figure 1:**
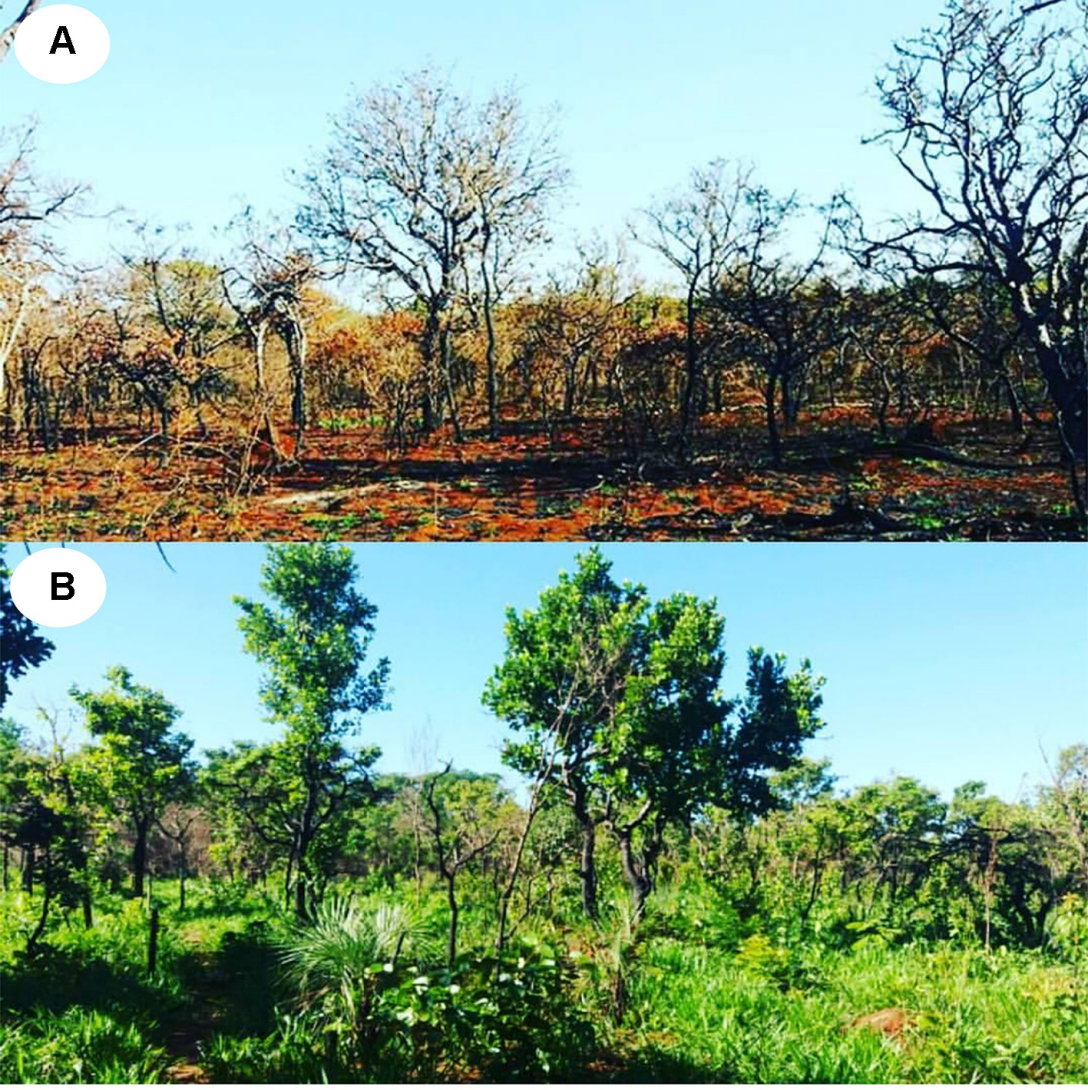
Fire tolerance of Cerrado tree species. The image shows the preservation unit of the University of Rio Verde 1 week after the fire incident **(A)**, and resprouting of the species 3 months after this event **(B)**.

Although this study was focused on the physiological implications of the variability in the relative investment of bark components at the branch level, the occurrence of an intense natural fire event, soon after the morpho-anatomical analyses, at the beginning of the dry season, allowed us to explore the resistance and resilience to fire of the species in that region (**Figure 1**). The classification of the intensity of the fire event was estimated as the char height (Hoffmann and Solbrig, 2003), based on measurements of fire marks on the bark of 30 trees across the experimental site, soon after the fire event.

To evaluate the resilience to fire of the selected species, soon after the fire event in the reserve (June 2017), five individuals of each species were monitored every 15 days for a period of 5 months, including the beginning of the rainy season. The criterion used to define fire-induced topkill was the absence of living branches or sprouting above 30 cm of the ground, throughout the sampling period (Hoffmann et al., 2009).

### Morphological Analyses

#### Bark Structure

Segments of approximately 10 mm were removed from the basal portion of the collected branches and photographed under a light microscope for subsequent analysis. The stem diameter (SD), the total bark thickness (TBT), the OB thickness (OBT), and the IB thickness (IBT) were measured using the ImageJ program following Rosell et al. (2017). In order to allow the comparison of the relative bark investment across species, we standardized the bark components by the SD. In this way, the relative thickness of the outer (RBT_outer_), inner (RBT_inner_), and total (RBT_total_) bark equaled two times the thickness of a given bark portion divided by the wood diameter of that branch segment (Rosell et al., 2014).

Given that fire resistance is mainly associated with bark properties of the main stem, in order to explore the bark insulating properties, we also measured the bark thickness of the main stem at 30 cm above the ground (TBT_30_), and expressed the relative investment of bark in this portion (RBT_30_) in the same way as described above. Measurements were made 3 months after the fire event in the region. Three 3 × 3 cm bark segments were removed from each individual down to the vascular cambium using a chisel, and the thickness was measured with calipers (Poorter et al., 2014). It is important to note that, in addition to the insulating against fire, this methodological approach also allowed to explore the coordination of the relative bark allocation between different plant organs.

#### Bark and Wood Density

The total bark density (*D*_bark_) and wood density (*D*_wood_) were determined in the same branch segments used for the bark structure measurements. The density was calculated by estimating the fresh tissue volume using the water displacement method. The samples were subsequently dried in an oven for 48 h at 72 °C, and the resulting dry mass was obtained using a precision balance (± 0.0001 g). The density (g cm^-3^) was calculated as the tissue dry mass/tissue volume (Markesteijn et al, 2011a).

#### Branch Growth Rate

To determine the branch growth rate (BGR), four terminal branches exposed to the sun from five individuals per species were marked and measured monthly using a measuring tape. The BGR values (mm day^-1^) represent the mean growth over 3 months of the rainy season (from February to April).

### Water Relations

#### Water Content of Leaves, Wood, and Bark

The water content of leaves (LWC), wood (WWC), and bark (BWC) were determined at midday, in segments from the same branches used for wood and bark morphological analyses, according to the following formula:

Water content (%)=100 x (fresh mass - dry mass)/dry mass

From these data, the percentage of stem water stored in the bark (%WinB) was calculated following Rosell et al. (2014), where:

%WinB=water in bark/(water in wood + water in bark) x 100

#### Stem Water Potential

The stem water potential at midday (Ψ_stem-md_) was estimated by measuring the water potential of a nontranspiring leaf (Mcculloh et al., 2014; Othman and Heerema, 2014). Between 09:00 and 10:00 h, one sun-exposed leaf from the same five individuals per species were wrapped in a plastic bag covered with aluminum foil to stop transpiration and allowed to equilibrate for at least 2 h (Othman and Heerema, 2014). After this equilibration time, around 12:00 and 13:00 h, the leaf was cut with a sharp razor blade, sealed in another plastic bag into which breath was exhaled, and stored in a cooler containing wet papers. The leaves were then transported to the lab where the Ψ_leaf-md_ was determined with a Scholander pressure chamber (Model SKPM 1405; Skye Instruments Ltd, Powys, UK).

#### Stem Hydraulic Conductivity

Sapwood-specific hydraulic conductivity (*K*_stem_) were determined for five individuals of each species, following the protocol proposed by Markesteijn et al. (2011a). Sun-exposed branches, next to the branches used for the wood and bark analyses, were harvested in the field, wrapped into dark plastic bags with wet paper towels inside, and transported to the laboratory. Once in the laboratory, the branches were recut under distilled water to avoid the introduction of new embolisms. For each species, the final size of the stem segment was higher than the length of the longest vessel. Distal ends were trimmed with a razor blade to clear any accidentally blocked vessel and approximately 1 cm of the bark at each side of the branch was removed. While submerged, the shaved end of the branch was connected to a tubing system attached to a pressurized reservoir (150 kPa) filled with a flow solution of 10 mmol KCL dissolved in degassed and filtered (0.2 µm) distilled water. The stems were flushed for 30 min to remove emboli and connected to a hydraulic flow apparatus (Sperry et al., 1988). An elevated water reservoir supplied the same flow solution to the stems, with the height quantified for each conductivity measurement to allow determination of pressure (approximately 5 kPa). The mass of the solution flowing per unit time through the segments was constantly monitored with an analytical balance. Hydraulic conductance (*K*_h_: in kg s^-1^ MPa^-1^) was calculated as:

$$Kh\;=\;\frac{\Delta V}{\left(\Delta P/\Delta X\right)}$$

where Δ*V* is the mass flow rate (kg s^-1^) and Δ*P* is the pressure drop (MPa) across a stem segment of length *X* (m). The sapwood area was estimated as the cross-sectional area of the upper distal end of the stem segment after bark removal, minus the cross-sectional area of pith. Sapwood-specific conductivity (*K*_stem_; kg m^-1^ s^-1^ MPa^-1^) was calculated by dividing *K*_h_ by the measured cross-sectional sapwood area.

### Gas Exchange

#### Net Carbon Assimilation

The net carbon assimilation rate *(A)* was determined in an open system under saturated light conditions (1,000 µmol photons m^-2^ s^-1^), temperature of 25°C and a CO_2_ partial pressure of 40 Pa using an infrared gas analyzer (LI-6800, LI-COR Inc., Nebraska, USA) equipped with a blue/red light source (model LI-6800, LI-COR). The *A* data were subsequently transformed into mass units (*A*_mass_) following Poorter and Bongers (2006). Gas exchange measurements were conducted between 08:00 and 11:00 am in sun-exposed, fully expanded leaves (five leaves per species), belonging to the same branches subsequently harvested for the determination of bark and wood traits.

### Statistical Analyses

Before analyses, species traits were log_10_-transformed if necessary, to improve homoscedasticity and normality. Pearson's linear correlation analyses were used to investigate the relationship between the structural variation in the bark components with the traits that reflect aspects of water and carbohydrate storage and transport among the species. Differences in the relative investment of bark components (RBT_inner_ and RBT_outer_) at the branch level was assessed by independent *t*-test with species as replicates. To reduce the dimensionality of the data set and to identify the variables that explained most of the total variation, a principal component analysis (PCA) was used to explore multivariate associations among bark traits with those related to water use and carbohydrate demand. All variables were log_10_-transformed before analysis, which is equivalent to the use of standardized data (z-transformation), and adequate for data with different measurement scales (Díaz et al., 2016). The amount of retained components for interpretation (**Figure 6**) was determined by the Horn's parallel analysis, using the package paran in R. All the analyses were performed in R v.3.5.1 (R Core Team, 2018).

### Phylogenetic Signal

As some of the studied species belong to the same family (**Table 1**), a certain degree of phylogenetical signal is expected, especially in morphological traits of bark and wood, as a result of the tendency of structural similarity among relatives. To test for phylogenetic signal on the studied traits we applied a randomization procedure based on phylogenetically independent contrasts (PICs) and the *K* statistics (Blomberg et al., 2008; Rosell et al., 2014), using the R package picante (Kembel et al., 2010). In order to conduct this analysis, a phylogenetic tree for the sampled species was constructed (**Figure S1**) using a mega-tree approach in the R package V.PhyloMaker (Jin and Qian, 2019). This mega-tree contains 74,533 vascular plant species and includes all plant families. Data were log_10_-transformed before analysis.

## Results

### Fire Resistance and Structural and Functional Variation in Stem Components Among Cerrado Species

**Figure 2:**
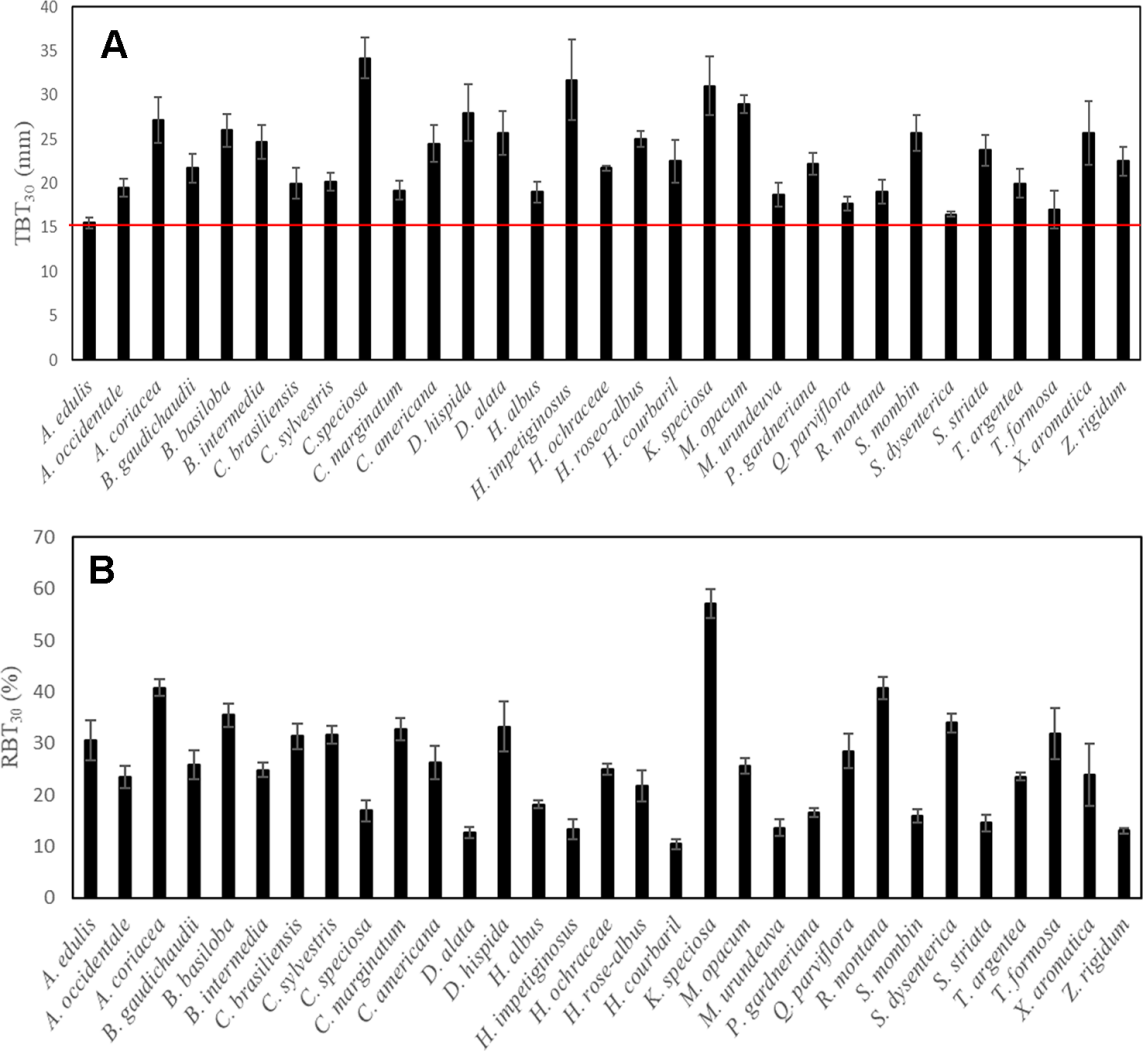
Variation among species in the total bark thickness at 30 cm from the ground (TBT_30_) **(A)**, and relative bark thickness of the main trunk (RBT_30_) **(B)**. Values are mean ± SE, n = 5. The red line indicates the threshold of bark thickness required for almost 100% of stem survival after fire in Cerrado, according to Hoffmann et al. (2009).

The surface fire event across the study site was classified as high intensity. The flame height was, on average, higher than 2 m (2.93 ± 0.29), and induced massive leaf abscission in all species analyzed. However, despite the high intensity of the surface fire event, no individual of the 31 species studied died after this event. In fact, 2 months after the fire, all plants had new leaves and/or signs of epicormic resprouting (**Figure 1**). It is important to note that among species, marked differences were observed in the TBT_30_ values (~2.2 times), one of the main traits associated with protection against fire. However, despite this great variability, all the species presented TBT_30_ values higher than 15 mm (**Figure 2**), further reinforcing that in this study only species with thick bark were examined.

**Figure 3:**
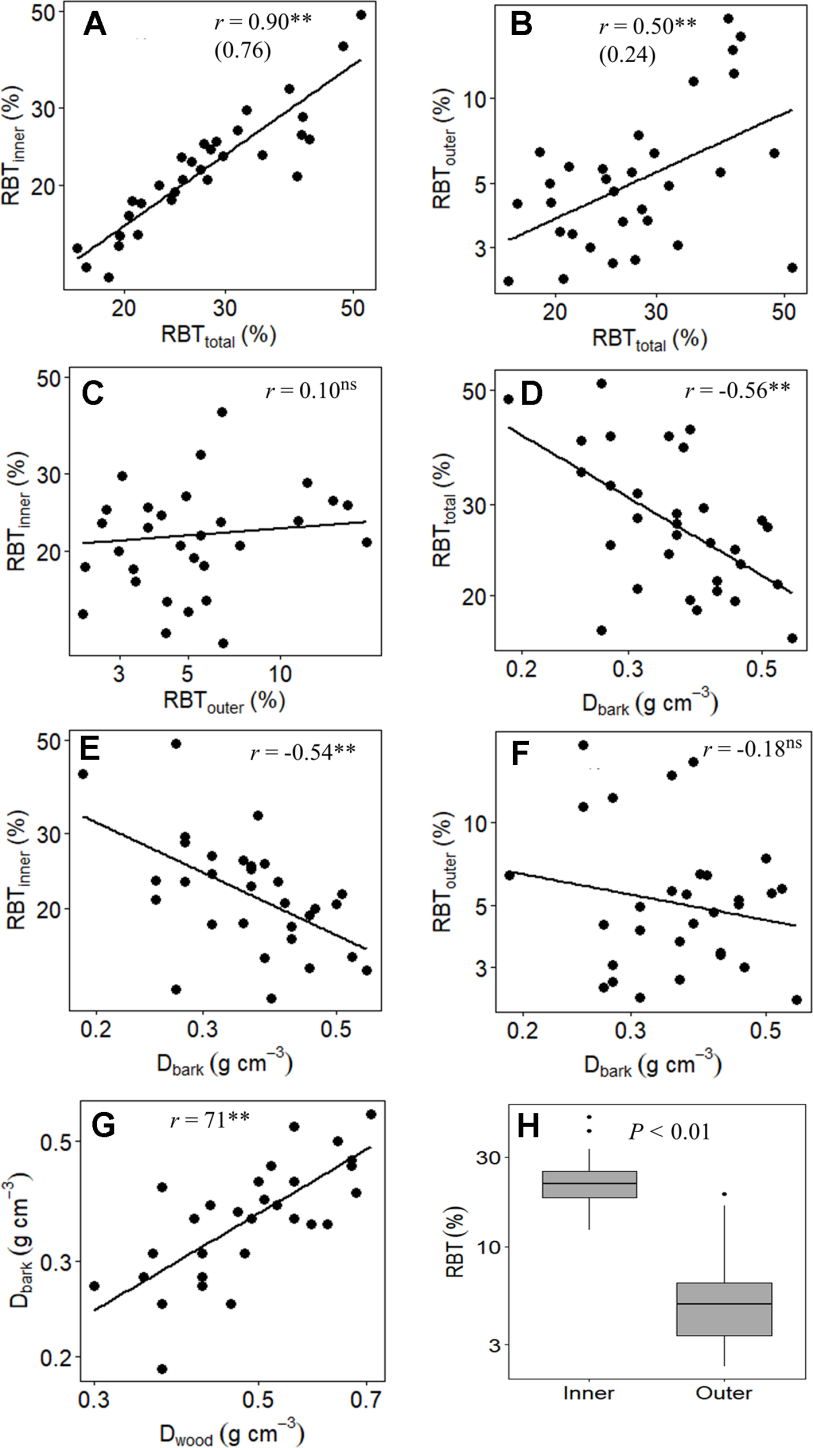
Branch-level relationship between **(A)** relative inner bark thickness (RBT_inner_) and relative total bark thickness (RBT_total_), **(B)** relative outer bark thickness (RBT_outer_) and RBT_total_, **(C)** RBT_inner_ and RBT_outer_, **(D)** RBT_total_ and the bark density (*D*_bark_), **(E)** RBT_inner_ and *D*_bark_, **(F)** RBT_outer_ and *D*_bark_, **(G)** *D*_bark_ and the wood density (*D*_wood_), and **(H)** boxplots of the relative thickness of inner and outer bark of 31 tree species from Brazilian Cerrado. Significance of Pearson correlation coefficients: ns, nonsignificant; *P ≤ 0.05; **P ≤ 0.01. In **(A** and **B)**, the slopes of the regression lines are given in parentheses.

At the branch level, the species showed a substantial structural divergence between the stem components (bark and wood) with the greatest variations observed for TBT, OBT, and IBT (**Tables 2** and **4**). The relative total investment in bark (RBT_total_) was directly related to the increase in SD (*r* = 0.67, *P* ≤ 0.01), which involved the contribution from both RBT_outer_ (*r* = 0.42, *P* ≤ 0.05) and RBT_inner_ (*r* = 0.55, *P* ≤ 0.01) (**Table 5**). However, despite the large variation in RBT_outer_ among species (~8-fold), RBT_inner_ was a better predictor of RBT_total_ variation (*r* = 0.90, *P* ≤ 0.01) when compared with OBT (*r* = 0.50, *P* ≤ 0.01) (**Tables 4** and **5**, **Figure 3**). The variation in bark structure, represented by *D*_bark_, was correlated only with changes in RBT_inner_ (*r* = -0.54, *P* ≤ 0.01) (**Figure 3**) since significant correlations were not found with RBT_outer_ (**Table 5**). Among the species analyzed, a significant relationship was found between the investment in bark and wood, as indicated by the high positive correlations between *D*_bark_ and *D*_wood_ (**Table 5**, **Figure 3**).

**Table 4:** Descriptive statistics and test for phylogenetic signal for bark, stem, water relations and growth traits for 31 tree species from Brazilian Cerrado.

	Mean	Min	Max	Ratio	*K*	*P*
**Bark traits**						
TBT_30_	23.1	15.5	34.2	2.21	0.25	0.14
RBT_30_	25.3	10.4	57.1	5.42	0.29	0.11
RBT_total_	29.0	17.0	51.7	3.04	0.20	0.25
RBT_outer_	6.07	2.29	19.1	8.32	0.14	0.54
RBT_inner_	22.4	12.4	49.0	3.98	0.34	0.07
*D*_bark_	0.37	0.19	0.56	2.90	0.31	0.06
**Stem traits**						
SD	1.06	0.74	1.52	2.05	0.12	0.71
*D*_wood_	0.50	0.30	0.71	2.33	0.42	0.02
**Water relations**						
BWC	64.2	51.0	77.4	1.52	0.18	0.34
WWC	53.1	40.4	66.0	1.63	0.33	0.04
LWC	57.7	46.0	67.4	1.47	0.31	0.09
%WinB	46.8	25.6	65.8	2.57	0.43	0.04
*Ψ*_stem-md_	-0.33	-0.75	-0.09	8.33	0.35	0.04
*K*_stem_	2.84	0.48	6.36	13.38	0.32	0.09
**Growth**						
*A*_mass_	126.40	53.83	283.7	5.27	0.14	0.61
BGR	0.14	0.04	0.34	8.50	0.31	0.05

The table shows overall mean (Mean), minimum (Min) and maximum (Max) species trait values, the rate of the maximum: minimum value (Ratio), the strength of phylogenetical signal (K) and the significance of the test detecting this signal (P).

**Table 5:** Pearson correlation between bark, stem and leaf traits among 31 tree species from Brazilian Cerrado.

	RBT_total_	RBT_outer_	RBT_inner_	RBT_30_	*D*_bark_	SD	*D*_wood_	BWC	WWC	LWC	%WinB	Ψ_stem-md_	*K*_stem_	BGR
**Bark traits**														
RBT_outer_	0.50**													
RBT_inner_	0.90**	0.10ns												
RBT_30_	0.44**	0.25ns	0.38*											
*D*_bark_	-0.56**	-0.18ns	-0.54**	-0.43**										
**Wood traits**														
SD	0.67**	0.42*	0.55**	0.27ns	-0.68**									
*D*_wood_	-0.38*	0.10ns	-0.45*	-0.35*	0.71**	-0.45**								
**Water relations**														
BWC	0.19ns	-0.28ns	0.35*	0.22ns	-0.79**	0.35*	-0.66**							
WWC	0.37*	-0.03ns	0.43*	0.39*	-0.70**	0.44**	-0.94**	0.64**						
LWC	0.10ns	-0.11ns	0.13ns	0.16ns	-0.65**	0.15ns	-0.69**	0.67**	0.67**					
%WinB	0.64**	0.25ns	0.63**	0.08ns	-0.37*	0.54**	-0.02ns	0.15ns	-0.02ns	-0.13ns				
Ψ_stem-md_	0.49**	0.18ns	0.49**	0.39*	-0.54**	0.48**	-0.52**	0.42*	0.56**	0.28ns	0.23ns			
*K*_stem_	0.05ns	-0.29ns	0.19ns	0.18ns	-0.53**	0.14ns	-0.71**	0.64**	0.69**	0.50**	0.10ns	-0.44**		
**Growth**														
BGR	0.20ns	0.13ns	0.16ns	0.14ns	-0.39*	0.44**	-0.25ns	0.38*	0.25ns	0.11ns	0.26ns	-0.23ns	0.03ns	
*A*_mass_	0.37*	0.22ns	0.35*	0.36*	-0.45**	0.50**	-0.50**	0.23ns	0.55**	0.04ns	0.09ns	-0.41*	0.58**	0.12ns

The table shows the correlation coefficients and their significance: ns, nonsignificant; *, P ≤ 0.05; **, P ≤ 0.01. Trait abbreviation as in **Tables 2** and **3**.

**Figure 4:**
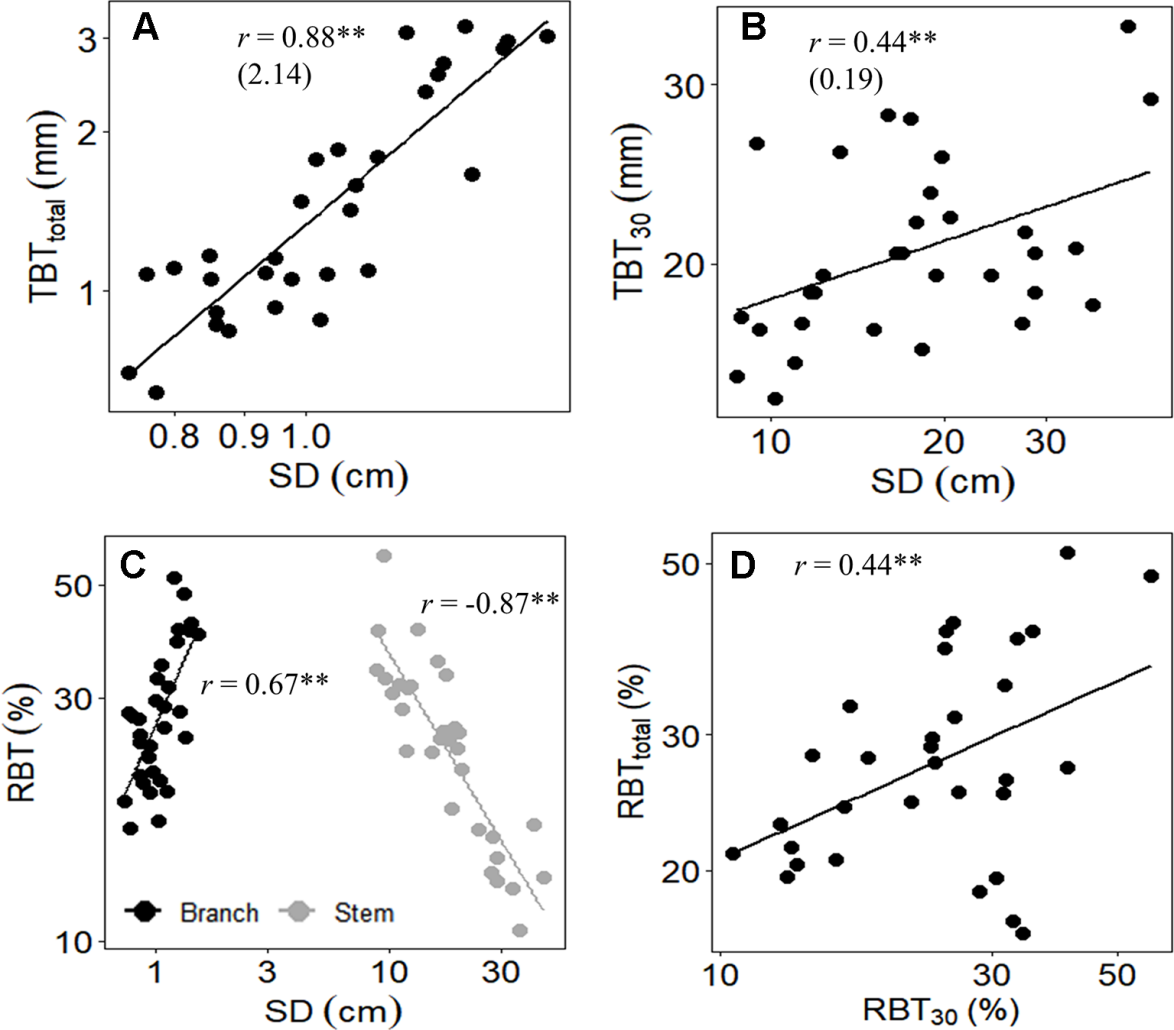
Relationships between **(A)** total bark thickness at 30 cm from the ground (TBT_30_) and the stem diameter (SD), **(B)** total bark thickness of branches (TBT_total_) and SD, **(C)** relative bark thickness of branches (RBT_total_, black circles) and main stem (RBT_30_, gray circles) with SD of both branches and stem, and **(D)** RBT_total_ and RBT_30_ of 31 tree species from Brazilian Cerrado. Significance of Pearson correlation coefficients: *P ≤ 0.05; **P ≤ 0.01. In **(A** and **B)**, the slopes of the regression lines are given in parentheses.

Among species, a similar pattern of bark allocation was found between branches and main stem, as denoted by the high positive correlations between RBT_total_ with RBT_30_ (**Table 5**, **Figure 4**). In both organs, variations in TBT were associated with increases in SD (**Figure 4**), but the relative bark investment between them followed different trends: at the main stem, RBT declined with the increment in SD, whereas at branches the opposite pattern was observed (**Figure 4**).

### Relationships Between Bark and Physiological Traits Associated With the Storage and Transport of Water

**Figure 5:**
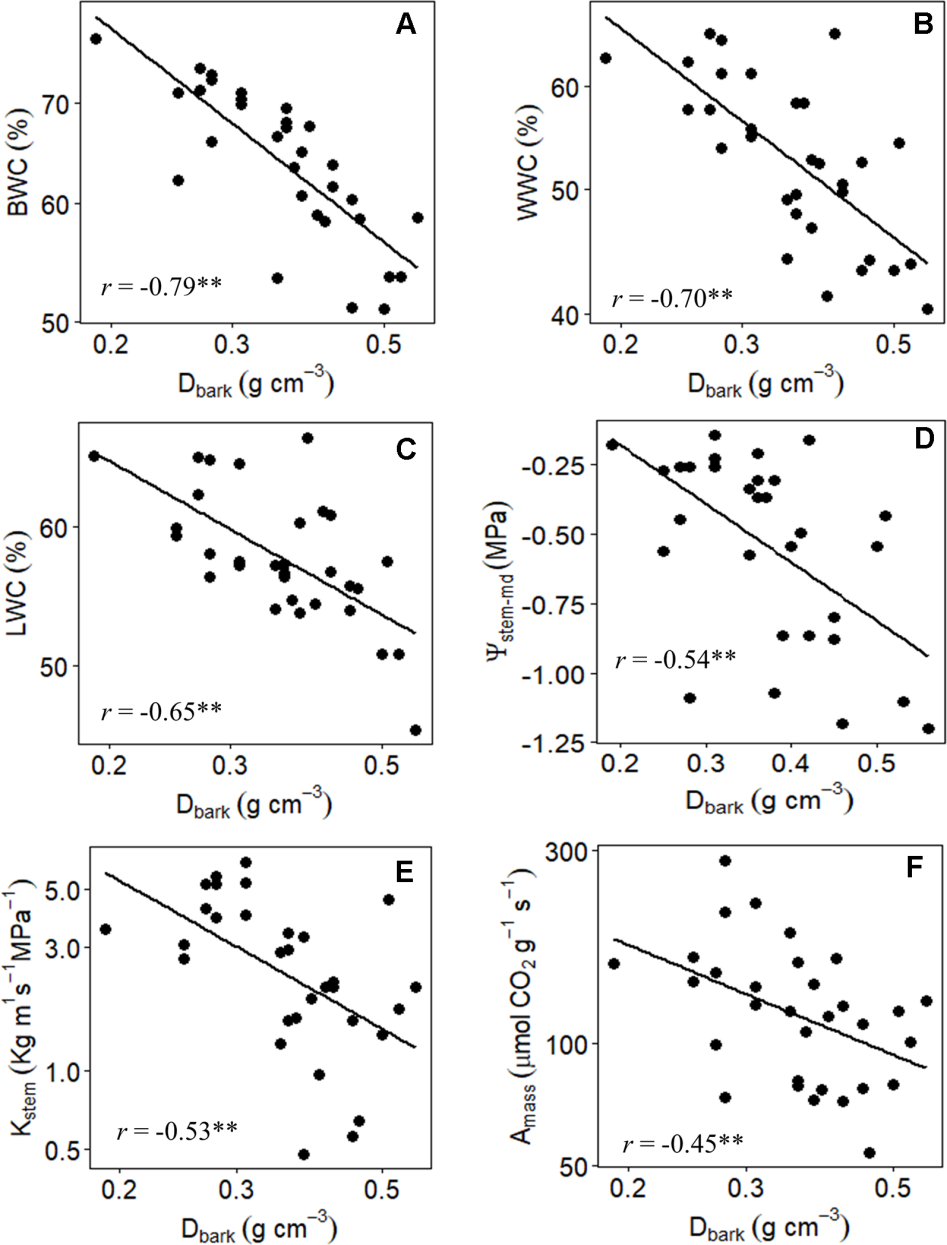
Branch-level relationship between **(A)** the bark water content (BWC), **(B)** wood water content (WWC), **(C)** leaf water content (LWC), **(D)** midday stem water potential (Ψ_stem-md_), **(E)** stem hydraulic conductivity, and **(F)** maximum CO_2_ assimilation on a leaf area basis (*A*_mass_), with bark density (*D*_bark_) of 31 tree species from Brazilian Cerrado. Significance of Pearson correlation coefficients: *P ≤ 0.05; **P ≤ 0.01.

Variation in the structural and functional properties of bark was directly related to traits that reflect different water use and conservation strategies, as well as carbon assimilation and growth (**Tables 2** and **3**). Regarding water relations, significant correlations were found between RBT_total_ with WWC, %WinB, and Ψ_stem-md_ (**Table 5**). However, by separating the bark into its main components, it was possible to observe that the processes of water storage and transport were only related to IBT, as evidenced by the highly significant correlations between RBT_inner_ and BWC, WWC, %WinB, and Ψ_stem-md_ (**Table 5**). In contrast, no significant correlation was found between RBT_outer_ and traits associated with water relations (**Table 5**). However, among all variables analyzed, *D*_bark_ was the one that best reflected the different strategies of water use and conservation of Cerrado plants, since it showed highly significant negative correlations with all traits associated with water relations (**Table 5**, **Figure 5**).

The large variation in the structural properties of bark was also directly related to the photosynthetic CO_2_ assimilation rate of the studied species. Indeed, *A*_mass_ values correlated positively with RBT_total_ and RBT_inner_, and negatively with *D*_bark_ (*r* = -0.45, *P* ≤ 0.01) (**Table 5**, **Figure 5**), indicating that plants with greater photosynthetic potential (**Table 3**) invested in a thicker and less dense bark (**Table 2**), characteristics that guarantee greater storage and transport capacity of water and other molecules. This increased storage and transport capacity was also directly related to higher growth rates, as observed from the significant negative correlation found between *D*_bark_ and BGR (*r* = -0.39, *P* ≤ 0.05) (**Table 5**). On the other hand, following the same pattern observed for water relations traits, no significative correlations were found between RBT_outer_ with traits associated with carbon assimilation and growth (**Table 5**).

### Bark and Its Association With Plant Growth and Water Use Strategies

**Figure 6:**
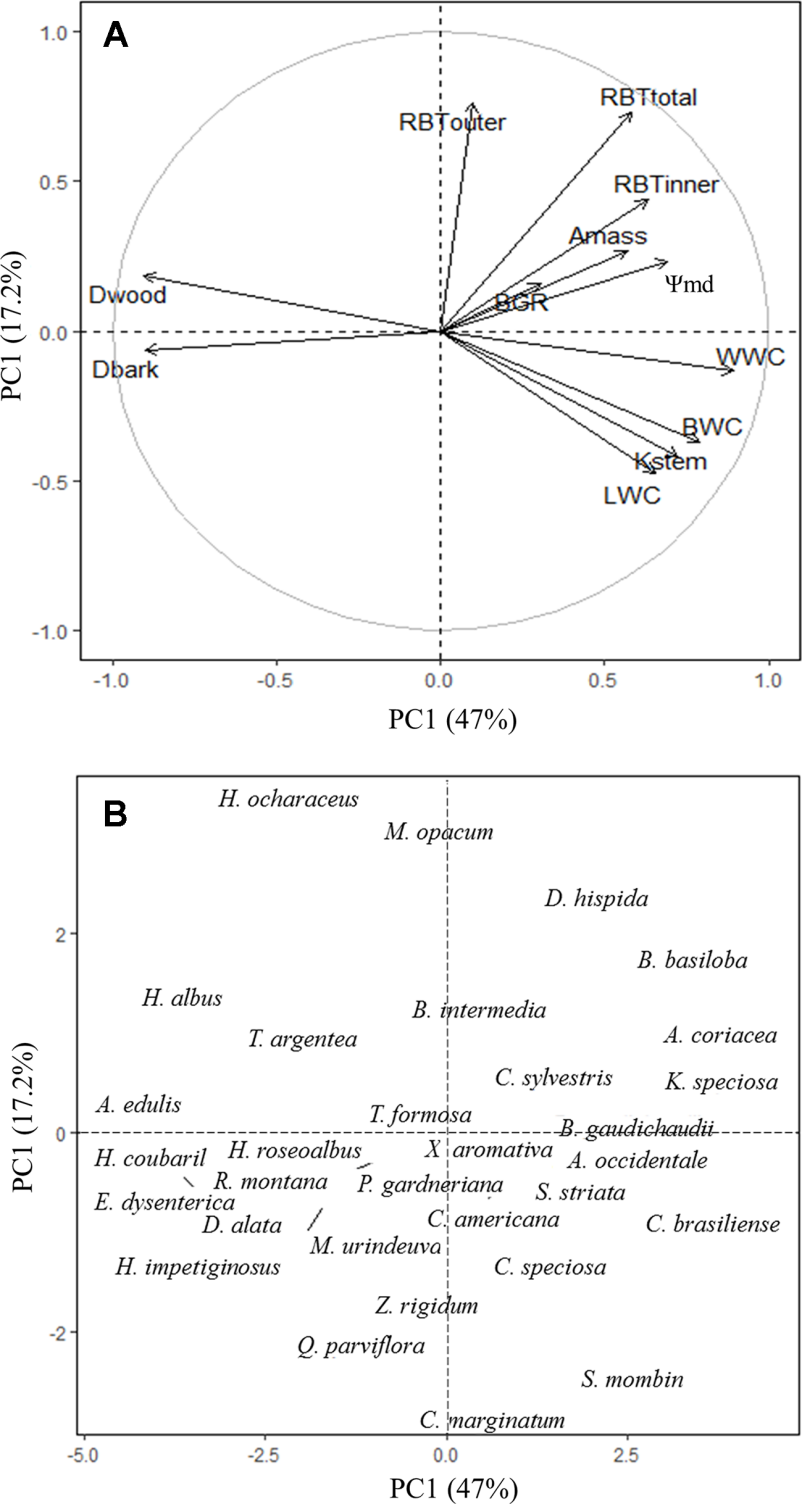
Principal component analysis (PCA) with the mean values of branch-level bark, wood, and leaf traits of 31 tree species from Brazilian Cerrado. Figures show the variation in trait scores **(A)** and species scores **(B)** along the first two PCA axes with the percentages of explained variation given. Trait abbreviation as in **Tables 2** and **3**.

The ecological implications of the structural and functional variation in bark among species were evaluated using a multivariate principal component analysis. The first two components explained 64.2% of the total variation in the data (**Figure 6**). The first component explained 47% of the total variation and clearly shows that species with higher investments in RBT_total_ and RBT_inner_, in addition to lower *D*_bark_, had greater water storage (BWC, WWC, LWC) and transport efficiency (*K*_stem_), tighter diurnal regulation of plant water status (higher Ψ_stem-md_), as well as greater photosynthetic potential (*A*_mass_) and growth rates (BGR) (**Figure 6**). The second component explained an additional 17.2% of the variation and shows that, along this axis, the species were primarily separated by their relative investment in bark (at the top of the second PCA axis), especially the outer portion of this structure (OB), and their capacity to store and transport water (at the bottom) (**Figure 6**).

### Phylogenetic Signal

Given the fact that some of the sampled species belong to the same family, we expected some phylogenetic signal on certain traits, especially the morphological ones. Despite our expectation, phylogenetic signal was not statistically significant for bark traits (**Table 4**), suggesting that variations in this structure are adjusted to local environmental conditions. A similar trend of absence of phylogenetic signal was also observed for most of the other structural and physiological traits analyzed. However, even on those traits in which a phylogenetic signal was detected, the magnitude of Blomberg's *K* tended to be considerably smaller than 1.0 (**Table 4**).

## Discussion

Cerrado plants are constantly exposed to two important selective pressures (fire and seasonal water restriction), which possibly shaped several structural and functional aspects of these species. In the present study, by analyzing 31 native Cerrado species, we have sought to identify the relative contribution of different bark components to the maintenance of central physiological processes. Our results suggest that the structural variation in the bark of Cerrado plants may reflect the role of this structure not only in the transport and storage of organic material but also in the regulation of plant water status. In this regard, we provide evidence that, at the branch level, the IB and OB exert contrasting ecological functional roles: IB was directly associated with the regulation of water status and photosynthetic capacity on a mass basis, whereas OB was possibly more strongly involved with defense against pathogen attack, mechanical support, and/or fire resistance.

### Bark and Its Central Role in the Protection Against Fire in Cerrado Plants

Although our study has focused on the physiological implications of the structural variation in bark components at the branch level, the occurrence of a natural fire event in the study area allowed us to explore the remarkable resistance and resilience to fire of Cerrado tree species. In fact, no individual of the 31 species studied died after the fire event (**Figure 1**). This ability to resist fire episodes appears to be directly related to the structural aspects of bark of the main stem, especially the thickness. Several studies have shown that the presence of a thicker bark is a determining factor in the higher postfire survival of Cerrado plants compared to species from other biomes (Hoffmann et al., 2009; Lawes et al., 2013; Rosell, 2016; Pellegrini et al., 2017). This greater investment in bark appears to be the probable explanation for the survival of the species studied here, as all species had TBT_30_ values (**Table 2**, **Figure 2**) above the threshold indicated as necessary to guarantee fire survival both in the Cerrado (Hoffmann et al., 2009), and in other savannas (Lawes et al., 2011), further reinforcing that this study examined only species with thick bark. However, although TBT_30_ represents a strong indicator of fire tolerance, which bark component would be more strongly associated with this insulating effect? All available evidence suggests the OB since changes in the thickness of this structure appear to represent one of the major ecological strategies in response to the selective pressure exerted by fire (Pausas, 2015a; Pausas, 2015b; Pausas et al., 2017; Rosell et al., 2017). These observations help to explain the greater relative investment in OB in plants inhabiting regions subject to frequent fire events, such as savannas, compared with plants in other biomes where the fire is not a constant (Lawes et al., 2013; Rosell et al., 2014; Pausas, 2015a).

### Determinants of Structural Variation in the Bark of Cerrado Species

In branches, the relative increase in TBT (RBT_total_) was directly associated with SD (**Table 5**). This relationship has been found in several studies and suggests that the investment in bark is due in large part to variation in plant size (Lawes et al., 2013; Poorter et al., 2014; Rosell, 2016; Rosell et al., 2017). However, it is important to note that this coordination between RBT_total_ and SD, in addition to representing a structural relationship, may also reflect different ecological strategies, particularly when we divide the bark into its main components (Rosell et al., 2014). In fact, although OBT explained a significant portion of the RBT_total_ variation among species, the contribution of IBT appears to be substantially more significant (**Figure 3**), as denoted by the much steeper slope of RBT_inner_ versus RBT_total_ than that of RBT_outer_ vs RBT_total_ (**Table 5**, **Figure 3**). When taken together, these results suggest that, in Cerrado trees, variations in RBT of branches are chiefly governed by higher investments in IB. However, it is important to note that although the studied species differed significantly in their relative investments in OBT and IBT (**Tables 2** and **4**), a significant correlation between these two bark structures was not found (**Figure 3**). This absence of correlation highlights the different ecological functions performed by the IB and OB (discussed below), as well as their different embryonic origins (Rosell, 2016).

Among species, the differential bark allocation observed at the branch level seems to have followed the same patterns of those in the main trunk, as denoted by the high positive correlation found between RBT_total_ and RBT_30_ (**Table 5**, **Figure 4**). This conserved allocation pattern of bark investment between branches and main trunk also seems to be extended to the physiological functions exerted by the bark, as the RBT_30_ and RBT_total_ were correlated to essentially all the same traits related to water use and conservation (**Table 5**). Thus, although our study has focused at the branch level, it is feasible to suggest that the bark of different plant structures possibly fulfills similar physiological functions. However, despite the apparent conserved physiological functions exerted by bark within different plant organs, the relative contribution of bark from each organ to these physiological functions seems to vary with their increment in size.

Although the TBT of both stems and branches had increased with SD, the relative bark investment on these organs differed substantially (**Figure 4**). At the main stem, RBT declined with increasing SD (**Figure 4**), a result already observed for several studies (Pausas, 2015a; Midgley et al., 2016), and which brings important insights related to bark investment and fire ecology. For example, some studies argued that this reduction in the relative investment in bark, as plants increase in size, typically occurs when a safe TBT is achieved (e.g., 15 mm for species from Cerrado, as in the case of this study), and thus further investment in bark would have less insulation value (Midgley et al., 2016). In addition to the reduction in bark allocation, the reduction in RBT with the increment in SD has also been related to bark shedding, since TBT is a function of bark allocation and retention (Midgley et al., 2016). On the other hand, contrary to what was observed on the main stem, at the branch level, a positive correlation was found between RBT with SD (**Figure 4**). Since variations in RBT_total_ among species were largely explained by the differential investment in IB (**Figure 3**), the increase in RBT with SD suggests that, in branches, there is a higher requirement of tissues associated with the IB rather than the OB (**Figure 3**). The ecological explanation for this higher allocation in IB, when compared to OB (**Table 4**, **Figure 3**), is possibly related to the role of the bark on branches in providing hydraulic and metabolic support for high demanding organs (as discussed below), especially the leaves. In fact, given that branches form a bridge that allows the traffic of several molecules into (e.g., water) and outside (e.g., carbohydrates) the leaves (Stroock et al., 2014), it is not surprising that this plant structure requires a higher investment in IB. Finally, considering the nature of fire events at Cerrado (e.g., surface-fires) (Hoffmann et al., 2009), this lower relative investment in OB is also expected at the branch level, since the main stem is the critical region in which the adaptation to fire is likely to be observed (Hoffmann et al., 2009; Lawes et al., 2011; Lawes et al., 2013).

### Structural and Functional Properties of the IB Are Related to Water Transport and Storage in Cerrado Plants

One of our predictions was that in addition to the selective pressure exerted by fire, the structural variation in the bark components of Cerrado plants also reflected different ecological strategies related to the maintenance and transport of water. This prediction appears to be confirmed since several significant correlations were found between bark properties and physiological traits associated with water relations (**Table 5**, **Figure 5**). In fact, our multivariate analysis showed that RBT_total_, a trait strongly associated with fire tolerance (Hoffmann et al., 2009; Pellegrini et al., 2017), was also able to capture several associations of the bark with processes of water storage and transport in the species studied here (**Table 5**, **Figure 5**). However, it is important to note that when we divide the bark into its main components, it is possible to observe that all those processes were associated only with the IB (**Table 5**). The absence of significative correlations between RBT_outer_ with any of the analyzed traits suggests that this portion of the bark may be associated with other physiological processes rather than water relations. In fact, it was already showed that, in addition to defense against fire, variation in the relative investment in OB are directly associated with mechanical support and defense against pathogen attack (Pausas, 2015a; 2015b), especially at branch levels (Rosell et al., 2014), as is the case of the present study.

Given the strong relationship between the IB and variables associated with water relations, it can be expected that the large variation in the relative investment in IB among the species (~4 times) (**Tables 2** and **4**) represents different strategies of water use and conservation. One of these strategies appears to involve water storage since the species with the highest RBT_inner_ values were those with the highest BWC and %WinB (**Table 5**). Similar results have been found by other studies, which have attributed this storage capacity to a large number of parenchyma cells present in the IB (Rosell et al., 2014; Pfautsch et al., 2015). However, this water storage potential, in addition to being a function of the amount of tissue invested (RBT_inner_), probably also depends on the structural properties of that tissue (density) (Rosell et al., 2014). In fact, tissues with a lower density tend to be composed of cells with thinner cell walls, which tends to increase their water storage capacity (Poorter et al., 2014). This observation helps to explain why *D*_bark_, among all variables analyzed, was the best predictor of bark water storage capacity (**Table 5**, **Figure 5**). In addition, the high negative correlation found between *D*_bark_ and RBT_inner_, and the absence of a significant correlation between *D*_bark_ and RBT_outer_ (**Table 5**), reinforces the evidence that this storage role is directly related to the IB properties (Rosell, 2016).

The structural and functional properties of bark were also related to the maintenance of the water status of other tissues. In fact, the species with the greatest capacity to store water in the bark (represented by a lower *D*_bark_ and a higher BWC) were the species with the highest quantities of water in the stem (WWC) and leaves (LWC) (**Table 5**, **Figure 5**). This higher storage capacity was also directly associated with lower variation in stem (Ψ_stem-md_) water status throughout the day (**Table 5**). These results reinforce the evidence that the water stored in bark can be used to sustain the transpiratory flux both daily and seasonally (Scholz et al., 2007; Poorter et al., 2014; Pfautsch et al., 2015). Apparently, this exchange of water between the IB and other tissues is mediated by radial cells, which form a bridge between the phloem and the xylem. In this way, the bark would act as a capacitor that, together with the main trunk, would form a large water reservoir (Pfautsch et al., 2015). In the present study, this relationship seems to be confirmed, since the structural properties of bark and trunk were equally important in explaining the variation in water status among species (**Table 5**, **Figures 4** and **5**). These results are in agreement with previous studies suggesting that the stem capacity to store water results from the interaction between bark and wood, and that none of these tissues likely operate in isolation (Niklas, 1999). Therefore, the high positive correlation found between *D*_bark_ and *D*_wood_ suggests that the coordination between the structural and functional properties of these two structures is an intrinsic characteristic of Cerrado plants, and that variation in the bark structure represents an important aspect of the water management strategies of these species.

The ability to avoid large variations in water status is of vital importance for the maintenance of various physiological processes (Trueba et al., 2017), particularly for species that inhabit regions that undergo periodic drought events such as the Cerrado (Scholz et al., 2007). Therefore, the ability to coordinate transpiration with uptake and transport of water among different tissues represents a preponderant factor to ensure the maintenance of central physiological process (e.g., photosynthesis) (Anderegg et al., 2015; Adams et al., 2017). In the present study, this coordination between water demand and supply appears to be confirmed. In fact, the highly significant correlations of *D*_bark_ and BWC with *K*_stem_ (**Table 5**, **Figure 5**) suggest that the potential of the IB to store and supply water to the xylem is a determining factor to allow an increase in the efficiency of long-distance transport of water (high stem hydraulic conductivity) in Cerrado plants. These results indicate that the high variability in water transport capacity among the studied species (~13 times) (**Tables 2** and **4**), in addition to being related to xylem characteristics, was probably also associated with morpho-anatomical adjustments in the structure of the IB (phloem), which reinforce the structural and functional coordination between bark and wood (**Figure 3**). In this regard, considering the high contribution of the IB to the overall TBT variation (**Table 5**, **Figure 3**), and that the functional properties of this bark component can affect both water storage and transport capacity, thus influencing the water status of leaves and wood (**Table 5**, **Figure 5**), the results of our branch-level study suggest that the relative investment in bark may also be adjusted to meet the hydric demands of other tissues, and thus reflect the contrasting water use strategies of the plants that inhabit this biome.

In addition to the role of bark in water transport, the large variation in IBT and *D*_bark_ across species (**Tables 2** and **4**) may also reflect another important ecological function performed by this structure: the transport of carbohydrates (Liesche et al., 2015). In fact, the IB includes the secondary phloem, which is the tissue responsible for transporting carbohydrates from the photosynthetic tissues to the rest of the plant (Rosell et al., 2014). In the present study, the coordination between the photosynthetic potential and the export capacity of assimilates could be visualized by the highly significant correlations of IBT and *D*_bark_ with *A*_mass_ (**Table 5**, **Figure 5**). These results suggest that plants with a higher photosynthetic potential should invest in a thicker and less dense bark, which are characteristics that enhance the ability to export molecules (Rosell et al., 2017). The synchronization between these processes is fundamental since an excess of carbohydrates in the source tissues can culminate in the retroinhibition of the photosynthetic process and consequently compromise growth (Scafaro et al., 2016). The highly negative correlations found between *D*_bark_ and BGR (**Table 5**) reinforces the occurrence of this synchronization and suggests that the bark structure in Cerrado plants, more specifically its inner portion, may also reflect the variability in metabolic demands as well as the different growth strategies among species of this domain.

### Tradeoffs Underlying the Differential Bark Investment Among Cerrado Tree Species

The differential bark investment among Cerrado tree species was able to reflect their contrasting strategies of water use and conservation and had a direct impact on the processes of carbon assimilation and growth. To allow high resource acquisition, fast-growing species (e.g., *Annona coriacea, Byrsonima basiloba, Caryocar brasiliensis*, and *Kielmeyera speciosa*) invested in soft tissues that maximize water storage (higher RBT_inner_ and lower *D*_bark_) and transport capacity (lower *D*_wood_ and higher *K*_stem_), allowing a better hydration of leaf tissues (higher LWC), which probably resulted in a higher stomatal conductance and, consequently, higher carbon assimilation (*A*_mass_) (**Figure 5**). The combination of high carbon assimilation and transport capacity with the investment in low dense tissues might also explain the higher BGR observed on those species (**Table 5**). However, although this strategy allows for higher resource acquisition, it may also expose the plants to a higher hydraulic risk, especially under drought conditions, a common situation in savannah biomes, like the Cerrado. In fact, several studies had already showed that, due to wider xylem vessels, species with less dense wood have higher hydraulic conductivity, which allows to higher growth rates but, at the same time, are more prone to cavitation (breakage of the water column) and, consequently, more vulnerable to drought (Santiago et al., 2004; Markesteijn et al., 2011a; Markesteijn et al., 2011b; Menezes-Silva et al., 2015). In contrast, slow-growing species (e.g., *Alibertia edulis, Handroanthus albus, Hymenaeae courbaril*, and *Stenocalyx dysenterica*) showed a more conservative strategy, characterized by the investment in more compact tissues, with lower water storage (lower RBT_inner_ and higher *D*_bark_) and transport capacity (lower *K*_stem_), but with a higher drought tolerance potential (higher *D*_wood_, probably due to narrow vessels) (**Figure 5**). Thus, given the high coordination found between xylem and phloem, and the relation of the IB in the regulation of the water status of wood and leaf tissues, our results suggest that the bark might represent a central component of the tradeoff between the water transport efficiency and safety among Cerrado tree species, with possible implications to the stem and leaf carbon economics (Wright et al., 2004; Chave et al., 2009). In this regard, further studies are needed to better understand the complexity of such tradeoffs, especially in the dry season, a situation in which the water stored in the bark represents a key component for the regulation of the plant global water status, and also to the flush of new organs (e.g., leaves) (Scholz et al., 2007).

## Conclusions

The results obtained in the present study provide a new perspective on the ecological implications of the structural and functional variation in the bark of Cerrado species. In addition to fire protection, the variability in the relative investment in IB and OB might also reflect the different strategies of water use and conservation, as well as metabolic demands among Cerrado tree species with contrasting growth strategies. We also provide strong evidence that different portions of bark are capable of performing contrasting ecological functions; at the branch level, the OB is probably more directly related to the role of mechanical support and/or defense against pathogen attack, whereas the IB is shaped primarily by the need for storage and transport of water, with direct impacts on carbon assimilation and growth. Overall, the present study significantly increases the knowledge regarding the ecophysiology of the plants that compose one of the largest and most diverse Brazilian biomes.

## Data Availability Statement

The raw data supporting the conclusions of this manuscript will be made available by the authors, without undue reservation, to any qualified researcher. All datasets generated for this study are included in the article/**Supplementary Material**.

## Author Contributions

PM-S and LL-L conceived the ideas and designed the experiment. PM-S, LL-L, LS, RA, and MA collected the data. LL-L, PM-S, FF, AC, FS, JG, HC, and AF analyzed the results. PM-S wrote the manuscript. All authors contributed critically to the drafts and gave final approval for publication.

## Funding

This study was financially supported by the Conselho Nacional de Desenvolvimento Científico e Tecnológico (CNPq), grant nº: 432264⁄2018-3, grant nº: 408083⁄2016, grant nº: 207920⁄2017-6; and by the Instituto Federal Goiano.

## Conflict of Interest

The authors declare that the research was conducted in the absence of any commercial or financial relationships that could be construed as a potential conflict of interest.
