# Supplementary figures and images for: A Structure Shaped by Fire, but Also Water: Ecological Consequences of the Variability in Bark Properties Across 31 Species From the Brazilian Cerrado
Source: Front Plant Sci. 2020 Jan 22;10:1718. doi: 10.3389/fpls.2019.01718 (PMC6987451; doi:10.3389/fpls.2019.01718)

## Supporting information

**Fig. S1.** Phylogenetic tree reconstructed for the studied species.

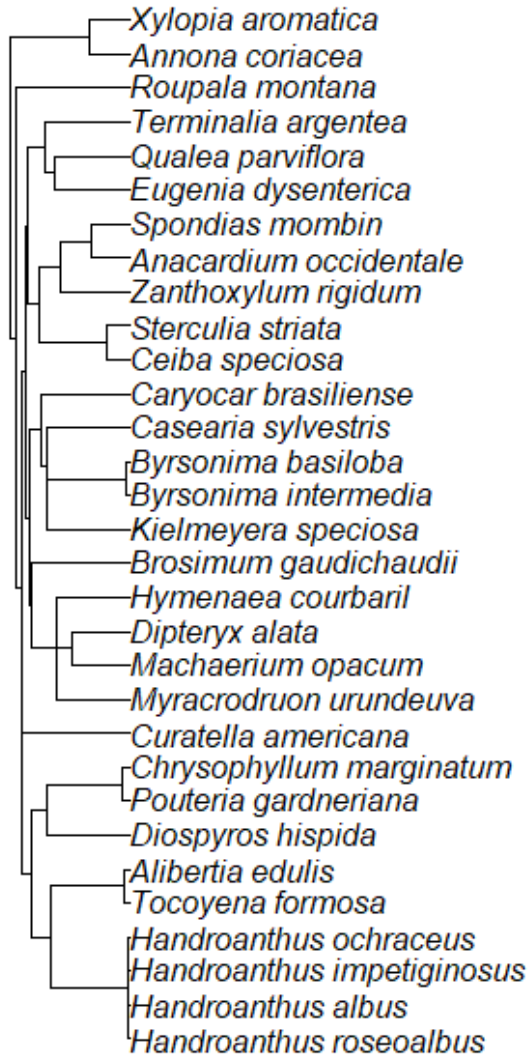

Supplement: Supplementary file 1 [file DataSheet_1.pdf]
